# Dimeth­yl(4′-pyridyl-2,2′:6′,2′′-terpyridine-κ^3^ *N* ^1^,*N* ^1′^,*N* ^1′′^)bis(thio­cyanato-κ*N*)tin(IV)

**DOI:** 10.1107/S1600536811001309

**Published:** 2011-01-15

**Authors:** Ezzatollah Najafi, Mostafa M. Amini, Seik Weng Ng

**Affiliations:** a Department of Chemistry, General Campus, Shahid Beheshti University, Tehran 1983963113, Iran; b Department of Chemistry, University of Malaya, 50603 Kuala Lumpur, Malaysia

## Abstract

The Sn atom in the title compound, [Sn(CH_3_)_2_(NCS)_2_(C_20_H_14_N_4_)], is *N*,*N*′,*N*′′-chelated by the terpyridine part of the *N*-heterocycle. The Sn atom exists in a *trans*-C_2_SnN_5_ penta­gonal–bipyramidal geometry [C—Sn—C = 173.66 (8)°] with the methyl groups in axial and the N atoms in equatorial positions.

## Related literature

For the dimethyl­tin dichloride–terpyridine adduct, see: Naik & Scheidt (1973 ▶).
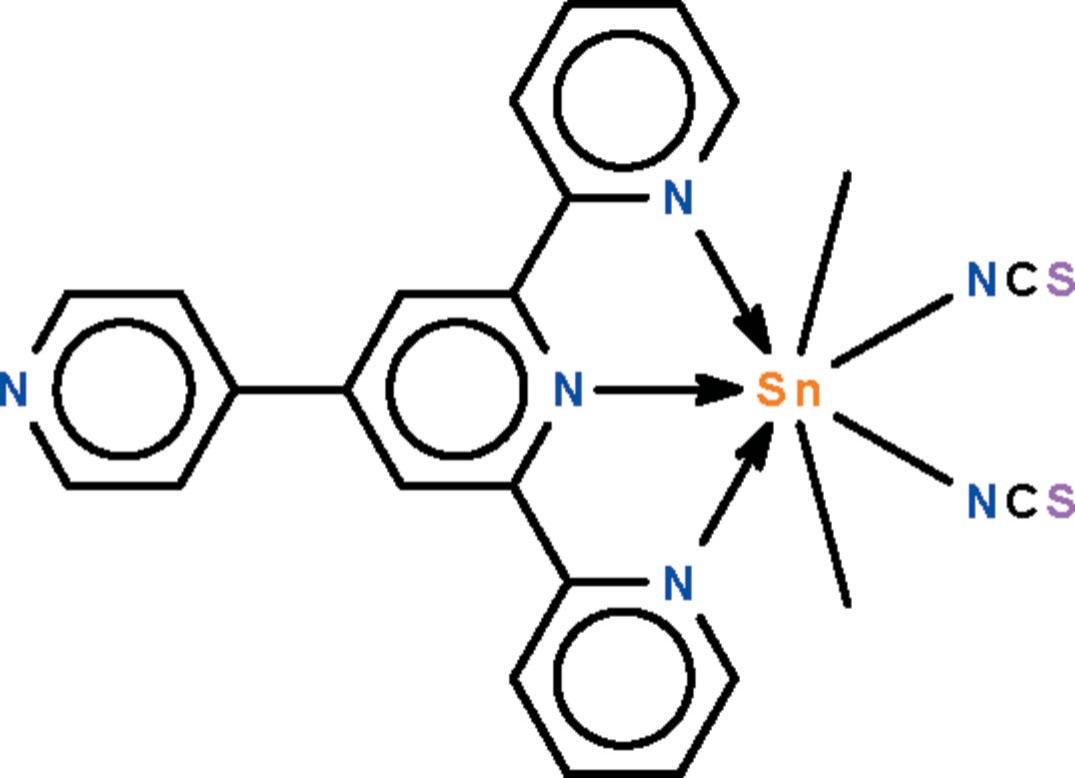


## Experimental

### 

#### Crystal data

[Sn(CH_3_)_2_(NCS)_2_(C_20_H_14_N_4_)]
*M* *_r_* = 575.27
Triclinic, 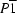

*a* = 9.3269 (3) Å
*b* = 10.5017 (3) Å
*c* = 13.1503 (4) Å
α = 66.814 (3)°
β = 87.665 (3)°
γ = 83.552 (2)°
*V* = 1176.52 (6) Å^3^
*Z* = 2
Mo *K*α radiation
μ = 1.29 mm^−1^
*T* = 100 K
0.20 × 0.20 × 0.10 mm

#### Data collection

Agilent Technologies SuperNova diffractometer with an Atlas detector
Absorption correction: multi-scan (*CrysAlis PRO*; Agilent Technologies, 2010 ▶) *T*_min_ = 0.783, *T*_max_ = 0.882
15704 measured reflections
5168 independent reflections
4728 reflections with *I* > 2σ(*I*)
*R*_int_ = 0.028

#### Refinement

*R*[*F* ^2^ > 2σ(*F* ^2^)] = 0.024
*wR*(*F* ^2^) = 0.060
*S* = 1.04
5168 reflections
300 parameters
H-atom parameters constrained
Δρ_max_ = 0.51 e Å^−3^
Δρ_min_ = −0.49 e Å^−3^

### 

Data collection: *CrysAlis PRO* (Agilent Technologies, 2010 ▶); cell refinement: *CrysAlis PRO*; data reduction: *CrysAlis PRO*; program(s) used to solve structure: *SHELXS97* (Sheldrick, 2008 ▶); program(s) used to refine structure: *SHELXL97* (Sheldrick, 2008 ▶); molecular graphics: *X-SEED* (Barbour, 2001 ▶); software used to prepare material for publication: *publCIF* (Westrip, 2010 ▶).

## Supplementary Material

Crystal structure: contains datablocks global, I. DOI: 10.1107/S1600536811001309/bt5461sup1.cif

Structure factors: contains datablocks I. DOI: 10.1107/S1600536811001309/bt5461Isup2.hkl

Additional supplementary materials:  crystallographic information; 3D view; checkCIF report

## Figures and Tables

**Fig. 1.**
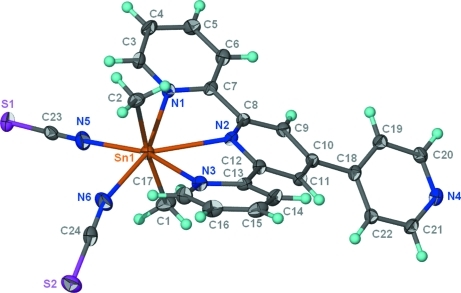
Anisotropic displacement ellipsoid plot (Barbour, 2001) of diisothiocyanatodimethyl(4'-pyridylterpyridine)tin at the 70% probability level; hydrogen atoms are drawn as spheres of arbitrary radius.

## supplementary crystallographic information

### Comment

Diorganotin dihalides/pseudohalides form a number of adducts with terpyridine and its derivatives. The dimethyltin diisocyanate forms an adduct with terpyridine itself (Naik & Scheidt, 1973); the ligand chelates to the tin atom through its three N atoms. The 4'-pyridylterpyridine has a similar seven-coordinate structure (Scheme I, Fig. 1). It also features the chelated tin atom in a seven-coordinate geometry.

### Experimental

Dimethyltin diisothiocyanate and 4'-pyridyl-2,2':6',2"-terpyridine (1 mmol) were loaded into a convection tube. The tube was filled with dry methanol and kept at 333 K. Colorless crystals were collected from the side arm after several days.

### Refinement

H-atoms were placed in calculated positions [C—H 0.95 to 0.98 Å, *U*_iso_(H) 1.2 to 1.5*U*_eq_(C)] and were included in the refinement in the riding model approximation.

### Crystal data

**Table:**

[Sn(CH_3_)_2_(NCS)_2_(C_20_H_14_N_4_)]	*Z* = 2
*M**_r_* = 575.27	*F*(000) = 576
Triclinic, *P*1	*D*_x_ = 1.624 Mg m^−^^3^
Hall symbol: -P 1	Mo *K*α radiation, λ = 0.71073 Å
*a* = 9.3269 (3) Å	Cell parameters from 10240 reflections
*b* = 10.5017 (3) Å	θ = 2.2–29.4°
*c* = 13.1503 (4) Å	µ = 1.29 mm^−^^1^
α = 66.814 (3)°	*T* = 100 K
β = 87.665 (3)°	Prism, colorless
γ = 83.552 (2)°	0.20 × 0.20 × 0.10 mm
*V* = 1176.52 (6) Å^3^	

### Data collection

**Table:**

Agilent Technologies SuperNova (Dual, Cu at zero) diffractometer with an Atlas detector	5168 independent reflections
Radiation source: SuperNova (Mo) X-ray Source	4728 reflections with *I* > 2σ(*I*)
Mirror	*R*_int_ = 0.028
Detector resolution: 10.4041 pixels mm^-1^	θ_max_ = 27.5°, θ_min_ = 2.2°
ω scans	*h* = −12→11
Absorption correction: multi-scan (*CrysAlis PRO*; Agilent Technologies, 2010)	*k* = −13→13
*T*_min_ = 0.783, *T*_max_ = 0.882	*l* = −17→16
15704 measured reflections	

### Refinement

**Table:**

Refinement on *F*^2^	Primary atom site location: structure-invariant direct methods
Least-squares matrix: full	Secondary atom site location: difference Fourier map
*R*[*F*^2^ > 2σ(*F*^2^)] = 0.024	Hydrogen site location: inferred from neighbouring sites
*wR*(*F*^2^) = 0.060	H-atom parameters constrained
*S* = 1.04	*w* = 1/[σ^2^(*F*_o_^2^) + (0.027*P*)^2^ + 0.2766*P*] where *P* = (*F*_o_^2^ + 2*F*_c_^2^)/3
5168 reflections	(Δ/σ)_max_ = 0.001
300 parameters	Δρ_max_ = 0.51 e Å^−^^3^
0 restraints	Δρ_min_ = −0.49 e Å^−^^3^

### Fractional atomic coordinates and isotropic or equivalent isotropic displacement parameters (Å^2^)

**Table:**

	*x*	*y*	*z*	*U*_iso_*/*U*_eq_	
Sn1	0.670329 (15)	0.378196 (13)	0.343774 (11)	0.01570 (6)	
S1	0.14249 (6)	0.55757 (6)	0.27483 (5)	0.02139 (12)	
S2	0.54381 (7)	0.07411 (6)	0.72375 (5)	0.02716 (14)	
N1	0.65080 (18)	0.52567 (17)	0.13746 (14)	0.0154 (4)	
N2	0.88281 (18)	0.34503 (16)	0.22361 (14)	0.0137 (4)	
N3	0.90522 (19)	0.26172 (17)	0.44304 (14)	0.0166 (4)	
N4	1.26459 (19)	−0.03774 (18)	−0.05850 (15)	0.0184 (4)	
N5	0.4323 (2)	0.45490 (19)	0.32114 (15)	0.0241 (4)	
N6	0.5991 (2)	0.2807 (2)	0.51867 (15)	0.0256 (4)	
C1	0.6332 (2)	0.1957 (2)	0.3225 (2)	0.0232 (5)	
H1A	0.6343	0.2139	0.2434	0.035*	
H1B	0.5391	0.1673	0.3536	0.035*	
H1C	0.7091	0.1210	0.3606	0.035*	
C2	0.7301 (2)	0.5579 (2)	0.35758 (18)	0.0201 (5)	
H2A	0.7350	0.5426	0.4359	0.030*	
H2B	0.6583	0.6369	0.3198	0.030*	
H2C	0.8247	0.5779	0.3235	0.030*	
C3	0.5515 (2)	0.6360 (2)	0.09447 (18)	0.0183 (4)	
H3	0.4899	0.6621	0.1439	0.022*	
C4	0.5342 (2)	0.7135 (2)	−0.01779 (18)	0.0200 (5)	
H4	0.4632	0.7915	−0.0447	0.024*	
C5	0.6229 (2)	0.6748 (2)	−0.08986 (17)	0.0185 (4)	
H5	0.6121	0.7244	−0.1675	0.022*	
C6	0.7274 (2)	0.5631 (2)	−0.04746 (17)	0.0166 (4)	
H6	0.7900	0.5355	−0.0956	0.020*	
C7	0.7396 (2)	0.4917 (2)	0.06618 (17)	0.0142 (4)	
C8	0.8551 (2)	0.3767 (2)	0.11653 (17)	0.0138 (4)	
C9	0.9287 (2)	0.3065 (2)	0.05728 (17)	0.0152 (4)	
H9	0.9077	0.3327	−0.0188	0.018*	
C10	1.0339 (2)	0.1968 (2)	0.11041 (17)	0.0146 (4)	
C11	1.0643 (2)	0.1662 (2)	0.22092 (17)	0.0155 (4)	
H11	1.1366	0.0935	0.2594	0.019*	
C12	0.9879 (2)	0.2429 (2)	0.27423 (17)	0.0142 (4)	
C13	1.0163 (2)	0.2199 (2)	0.39065 (17)	0.0158 (4)	
C14	1.1508 (2)	0.1658 (2)	0.43977 (18)	0.0186 (5)	
H14	1.2260	0.1346	0.4015	0.022*	
C15	1.1733 (2)	0.1583 (2)	0.54597 (18)	0.0206 (5)	
H15	1.2647	0.1238	0.5811	0.025*	
C16	1.0606 (2)	0.2018 (2)	0.59894 (18)	0.0214 (5)	
H16	1.0731	0.1974	0.6716	0.026*	
C17	0.9282 (2)	0.2523 (2)	0.54579 (18)	0.0202 (5)	
H17	0.8509	0.2813	0.5837	0.024*	
C18	1.1117 (2)	0.1161 (2)	0.05122 (17)	0.0148 (4)	
C19	1.1493 (2)	0.1789 (2)	−0.05975 (17)	0.0165 (4)	
H19	1.1225	0.2753	−0.1005	0.020*	
C20	1.2262 (2)	0.0990 (2)	−0.11010 (17)	0.0172 (4)	
H20	1.2531	0.1439	−0.1853	0.021*	
C21	1.2265 (2)	−0.0968 (2)	0.04727 (18)	0.0176 (4)	
H21	1.2523	−0.1940	0.0853	0.021*	
C22	1.1520 (2)	−0.0258 (2)	0.10523 (17)	0.0156 (4)	
H22	1.1286	−0.0733	0.1809	0.019*	
C23	0.3116 (2)	0.4974 (2)	0.30293 (17)	0.0173 (4)	
C24	0.5756 (2)	0.1937 (2)	0.60383 (18)	0.0198 (5)	

### Atomic displacement parameters (Å^2^)

**Table:**

	*U*^11^	*U*^22^	*U*^33^	*U*^12^	*U*^13^	*U*^23^
Sn1	0.01512 (9)	0.01554 (8)	0.01612 (9)	−0.00096 (6)	0.00146 (6)	−0.00616 (6)
S1	0.0158 (3)	0.0253 (3)	0.0245 (3)	0.0018 (2)	0.0006 (2)	−0.0123 (2)
S2	0.0260 (3)	0.0268 (3)	0.0212 (3)	−0.0053 (2)	0.0007 (3)	−0.0008 (2)
N1	0.0135 (9)	0.0140 (8)	0.0186 (9)	−0.0003 (7)	0.0013 (7)	−0.0068 (7)
N2	0.0137 (8)	0.0124 (8)	0.0152 (9)	0.0003 (7)	−0.0017 (7)	−0.0059 (7)
N3	0.0198 (9)	0.0161 (9)	0.0148 (9)	−0.0019 (7)	0.0007 (8)	−0.0069 (7)
N4	0.0171 (9)	0.0202 (9)	0.0200 (10)	0.0007 (7)	0.0010 (8)	−0.0107 (8)
N5	0.0177 (10)	0.0272 (10)	0.0232 (10)	0.0003 (8)	0.0035 (8)	−0.0065 (8)
N6	0.0252 (11)	0.0283 (10)	0.0178 (10)	−0.0040 (8)	0.0061 (9)	−0.0034 (8)
C1	0.0242 (12)	0.0159 (10)	0.0304 (13)	−0.0034 (9)	−0.0040 (10)	−0.0091 (10)
C2	0.0256 (12)	0.0136 (10)	0.0220 (12)	−0.0013 (9)	0.0013 (10)	−0.0082 (9)
C3	0.0154 (11)	0.0178 (10)	0.0224 (12)	0.0005 (8)	0.0033 (9)	−0.0096 (9)
C4	0.0172 (11)	0.0154 (10)	0.0244 (12)	0.0043 (9)	−0.0036 (10)	−0.0057 (9)
C5	0.0198 (11)	0.0189 (10)	0.0141 (11)	0.0002 (9)	−0.0040 (9)	−0.0040 (9)
C6	0.0170 (11)	0.0184 (10)	0.0166 (11)	−0.0009 (8)	−0.0001 (9)	−0.0094 (9)
C7	0.0133 (10)	0.0127 (9)	0.0185 (11)	−0.0009 (8)	−0.0019 (9)	−0.0081 (8)
C8	0.0125 (10)	0.0120 (9)	0.0164 (10)	−0.0012 (8)	−0.0001 (8)	−0.0049 (8)
C9	0.0155 (10)	0.0155 (10)	0.0148 (10)	−0.0016 (8)	0.0002 (9)	−0.0064 (8)
C10	0.0133 (10)	0.0142 (9)	0.0177 (11)	−0.0030 (8)	0.0028 (9)	−0.0076 (8)
C11	0.0141 (10)	0.0130 (9)	0.0174 (11)	0.0004 (8)	0.0003 (9)	−0.0041 (8)
C12	0.0124 (10)	0.0153 (10)	0.0151 (10)	−0.0014 (8)	0.0003 (8)	−0.0062 (8)
C13	0.0182 (11)	0.0111 (9)	0.0171 (11)	−0.0009 (8)	0.0005 (9)	−0.0046 (8)
C14	0.0173 (11)	0.0178 (10)	0.0199 (11)	0.0039 (9)	−0.0038 (9)	−0.0078 (9)
C15	0.0208 (11)	0.0162 (10)	0.0226 (12)	0.0007 (9)	−0.0102 (10)	−0.0049 (9)
C16	0.0287 (12)	0.0208 (11)	0.0155 (11)	−0.0032 (10)	−0.0044 (10)	−0.0075 (9)
C17	0.0230 (12)	0.0212 (11)	0.0181 (11)	−0.0026 (9)	0.0011 (10)	−0.0095 (9)
C18	0.0120 (10)	0.0163 (10)	0.0186 (11)	−0.0013 (8)	0.0001 (9)	−0.0097 (9)
C19	0.0156 (10)	0.0154 (10)	0.0179 (11)	0.0001 (8)	−0.0007 (9)	−0.0063 (8)
C20	0.0160 (11)	0.0203 (10)	0.0147 (11)	−0.0020 (9)	0.0011 (9)	−0.0063 (9)
C21	0.0153 (10)	0.0146 (10)	0.0224 (12)	0.0014 (8)	−0.0006 (9)	−0.0075 (9)
C22	0.0133 (10)	0.0172 (10)	0.0154 (10)	−0.0006 (8)	0.0007 (9)	−0.0057 (8)
C23	0.0216 (12)	0.0164 (10)	0.0131 (10)	−0.0041 (9)	0.0058 (9)	−0.0050 (8)
C24	0.0131 (10)	0.0261 (11)	0.0228 (12)	0.0001 (9)	−0.0008 (9)	−0.0129 (10)

### Geometric parameters (Å, °)

**Table:**

Sn1—C2	2.102 (2)	C5—C6	1.382 (3)
Sn1—C1	2.110 (2)	C5—H5	0.9500
Sn1—N6	2.2258 (18)	C6—C7	1.386 (3)
Sn1—N5	2.2645 (19)	C6—H6	0.9500
Sn1—N3	2.5246 (17)	C7—C8	1.481 (3)
Sn1—N1	2.5418 (17)	C8—C9	1.385 (3)
Sn1—N2	2.5630 (17)	C9—C10	1.395 (3)
S1—C23	1.632 (2)	C9—H9	0.9500
S2—C24	1.624 (2)	C10—C11	1.393 (3)
N1—C3	1.343 (3)	C10—C18	1.478 (3)
N1—C7	1.351 (3)	C11—C12	1.387 (3)
N2—C8	1.342 (3)	C11—H11	0.9500
N2—C12	1.345 (3)	C12—C13	1.483 (3)
N3—C17	1.340 (3)	C13—C14	1.389 (3)
N3—C13	1.349 (3)	C14—C15	1.391 (3)
N4—C21	1.332 (3)	C14—H14	0.9500
N4—C20	1.337 (3)	C15—C16	1.372 (3)
N5—C23	1.161 (3)	C15—H15	0.9500
N6—C24	1.164 (3)	C16—C17	1.388 (3)
C1—H1A	0.9800	C16—H16	0.9500
C1—H1B	0.9800	C17—H17	0.9500
C1—H1C	0.9800	C18—C22	1.389 (3)
C2—H2A	0.9800	C18—C19	1.393 (3)
C2—H2B	0.9800	C19—C20	1.385 (3)
C2—H2C	0.9800	C19—H19	0.9500
C3—C4	1.383 (3)	C20—H20	0.9500
C3—H3	0.9500	C21—C22	1.382 (3)
C4—C5	1.383 (3)	C21—H21	0.9500
C4—H4	0.9500	C22—H22	0.9500
			
C2—Sn1—C1	173.66 (8)	C7—C6—C5	119.20 (19)
C2—Sn1—N6	95.00 (8)	C7—C6—H6	120.4
C1—Sn1—N6	88.97 (8)	C5—C6—H6	120.4
C2—Sn1—N5	94.55 (8)	N1—C7—C6	122.26 (18)
C1—Sn1—N5	90.97 (8)	N1—C7—C8	116.12 (18)
N6—Sn1—N5	80.75 (7)	C6—C7—C8	121.57 (18)
C2—Sn1—N3	84.97 (7)	N2—C8—C9	122.46 (18)
C1—Sn1—N3	91.04 (7)	N2—C8—C7	115.30 (17)
N6—Sn1—N3	77.94 (6)	C9—C8—C7	122.25 (18)
N5—Sn1—N3	158.55 (6)	C8—C9—C10	119.37 (19)
C2—Sn1—N1	85.50 (7)	C8—C9—H9	120.3
C1—Sn1—N1	92.67 (7)	C10—C9—H9	120.3
N6—Sn1—N1	158.04 (6)	C11—C10—C9	117.91 (18)
N5—Sn1—N1	77.33 (6)	C11—C10—C18	120.97 (18)
N3—Sn1—N1	123.89 (5)	C9—C10—C18	121.12 (19)
C2—Sn1—N2	97.06 (7)	C12—C11—C10	119.39 (19)
C1—Sn1—N2	76.72 (7)	C12—C11—H11	120.3
N6—Sn1—N2	138.11 (6)	C10—C11—H11	120.3
N5—Sn1—N2	137.58 (6)	N2—C12—C11	122.32 (19)
N3—Sn1—N2	63.44 (5)	N2—C12—C13	114.86 (17)
N1—Sn1—N2	63.18 (5)	C11—C12—C13	122.81 (18)
C3—N1—C7	117.48 (17)	N3—C13—C14	122.62 (19)
C3—N1—Sn1	122.53 (13)	N3—C13—C12	115.37 (18)
C7—N1—Sn1	119.96 (12)	C14—C13—C12	121.93 (19)
C8—N2—C12	118.48 (17)	C15—C14—C13	118.8 (2)
C8—N2—Sn1	117.17 (13)	C15—C14—H14	120.6
C12—N2—Sn1	116.78 (13)	C13—C14—H14	120.6
C17—N3—C13	117.84 (18)	C16—C15—C14	118.5 (2)
C17—N3—Sn1	120.77 (14)	C16—C15—H15	120.7
C13—N3—Sn1	121.07 (13)	C14—C15—H15	120.7
C21—N4—C20	116.62 (17)	C15—C16—C17	119.7 (2)
C23—N5—Sn1	176.01 (17)	C15—C16—H16	120.2
C24—N6—Sn1	159.06 (18)	C17—C16—H16	120.2
Sn1—C1—H1A	109.5	N3—C17—C16	122.5 (2)
Sn1—C1—H1B	109.5	N3—C17—H17	118.7
H1A—C1—H1B	109.5	C16—C17—H17	118.7
Sn1—C1—H1C	109.5	C22—C18—C19	117.38 (18)
H1A—C1—H1C	109.5	C22—C18—C10	120.88 (18)
H1B—C1—H1C	109.5	C19—C18—C10	121.73 (18)
Sn1—C2—H2A	109.5	C20—C19—C18	119.26 (19)
Sn1—C2—H2B	109.5	C20—C19—H19	120.4
H2A—C2—H2B	109.5	C18—C19—H19	120.4
Sn1—C2—H2C	109.5	N4—C20—C19	123.52 (19)
H2A—C2—H2C	109.5	N4—C20—H20	118.2
H2B—C2—H2C	109.5	C19—C20—H20	118.2
N1—C3—C4	123.57 (19)	N4—C21—C22	124.19 (19)
N1—C3—H3	118.2	N4—C21—H21	117.9
C4—C3—H3	118.2	C22—C21—H21	117.9
C3—C4—C5	118.28 (19)	C21—C22—C18	119.01 (19)
C3—C4—H4	120.9	C21—C22—H22	120.5
C5—C4—H4	120.9	C18—C22—H22	120.5
C6—C5—C4	119.17 (19)	N5—C23—S1	178.9 (2)
C6—C5—H5	120.4	N6—C24—S2	178.9 (2)
C4—C5—H5	120.4		
			
C2—Sn1—N1—C3	−65.95 (16)	Sn1—N1—C7—C8	−6.5 (2)
C1—Sn1—N1—C3	120.14 (17)	C5—C6—C7—N1	1.4 (3)
N6—Sn1—N1—C3	26.3 (3)	C5—C6—C7—C8	−176.24 (19)
N5—Sn1—N1—C3	29.74 (16)	C12—N2—C8—C9	1.2 (3)
N3—Sn1—N1—C3	−146.81 (15)	Sn1—N2—C8—C9	−147.48 (16)
N2—Sn1—N1—C3	−166.20 (17)	C12—N2—C8—C7	−179.06 (17)
C2—Sn1—N1—C7	116.06 (16)	Sn1—N2—C8—C7	32.2 (2)
C1—Sn1—N1—C7	−57.86 (16)	N1—C7—C8—N2	−17.1 (3)
N6—Sn1—N1—C7	−151.74 (18)	C6—C7—C8—N2	160.63 (18)
N5—Sn1—N1—C7	−148.25 (16)	N1—C7—C8—C9	162.59 (18)
N3—Sn1—N1—C7	35.19 (16)	C6—C7—C8—C9	−19.7 (3)
N2—Sn1—N1—C7	15.81 (13)	N2—C8—C9—C10	1.2 (3)
C2—Sn1—N2—C8	−106.42 (14)	C7—C8—C9—C10	−178.43 (18)
C1—Sn1—N2—C8	74.85 (14)	C8—C9—C10—C11	−2.5 (3)
N6—Sn1—N2—C8	147.95 (14)	C8—C9—C10—C18	177.98 (18)
N5—Sn1—N2—C8	−1.71 (18)	C9—C10—C11—C12	1.3 (3)
N3—Sn1—N2—C8	172.83 (15)	C18—C10—C11—C12	−179.16 (18)
N1—Sn1—N2—C8	−25.11 (13)	C8—N2—C12—C11	−2.5 (3)
C2—Sn1—N2—C12	104.32 (15)	Sn1—N2—C12—C11	146.34 (16)
C1—Sn1—N2—C12	−74.41 (15)	C8—N2—C12—C13	176.96 (17)
N6—Sn1—N2—C12	−1.31 (19)	Sn1—N2—C12—C13	−34.2 (2)
N5—Sn1—N2—C12	−150.97 (14)	C10—C11—C12—N2	1.2 (3)
N3—Sn1—N2—C12	23.57 (13)	C10—C11—C12—C13	−178.18 (18)
N1—Sn1—N2—C12	−174.37 (16)	C17—N3—C13—C14	1.2 (3)
C2—Sn1—N3—C17	62.31 (16)	Sn1—N3—C13—C14	174.64 (15)
C1—Sn1—N3—C17	−122.63 (16)	C17—N3—C13—C12	−175.43 (18)
N6—Sn1—N3—C17	−33.91 (16)	Sn1—N3—C13—C12	−2.0 (2)
N5—Sn1—N3—C17	−27.3 (3)	N2—C12—C13—N3	24.1 (3)
N1—Sn1—N3—C17	143.45 (14)	C11—C12—C13—N3	−156.48 (19)
N2—Sn1—N3—C17	162.79 (17)	N2—C12—C13—C14	−152.59 (19)
C2—Sn1—N3—C13	−110.95 (16)	C11—C12—C13—C14	26.9 (3)
C1—Sn1—N3—C13	64.12 (16)	N3—C13—C14—C15	−2.0 (3)
N6—Sn1—N3—C13	152.84 (16)	C12—C13—C14—C15	174.45 (19)
N5—Sn1—N3—C13	159.42 (17)	C13—C14—C15—C16	1.4 (3)
N1—Sn1—N3—C13	−29.80 (17)	C14—C15—C16—C17	−0.2 (3)
N2—Sn1—N3—C13	−10.47 (14)	C13—N3—C17—C16	0.1 (3)
C2—Sn1—N6—C24	−150.2 (5)	Sn1—N3—C17—C16	−173.39 (15)
C1—Sn1—N6—C24	24.8 (5)	C15—C16—C17—N3	−0.5 (3)
N5—Sn1—N6—C24	116.0 (5)	C11—C10—C18—C22	35.8 (3)
N3—Sn1—N6—C24	−66.5 (5)	C9—C10—C18—C22	−144.7 (2)
N1—Sn1—N6—C24	119.4 (5)	C11—C10—C18—C19	−143.3 (2)
N2—Sn1—N6—C24	−43.8 (5)	C9—C10—C18—C19	36.3 (3)
C7—N1—C3—C4	1.3 (3)	C22—C18—C19—C20	−1.2 (3)
Sn1—N1—C3—C4	−176.70 (15)	C10—C18—C19—C20	177.93 (19)
N1—C3—C4—C5	0.7 (3)	C21—N4—C20—C19	−0.7 (3)
C3—C4—C5—C6	−1.7 (3)	C18—C19—C20—N4	1.5 (3)
C4—C5—C6—C7	0.7 (3)	C20—N4—C21—C22	−0.4 (3)
C3—N1—C7—C6	−2.4 (3)	N4—C21—C22—C18	0.7 (3)
Sn1—N1—C7—C6	175.74 (15)	C19—C18—C22—C21	0.1 (3)
C3—N1—C7—C8	175.36 (18)	C10—C18—C22—C21	−178.98 (19)

## References

[bb1] Agilent Technologies (2010). *CrysAlis PRO* Agilent Technologies, Yarnton, England.

[bb2] Barbour, L. J. (2001). *J. Supramol. Chem.* **1**, 189–191.

[bb3] Naik, D. N. & Scheidt, W. R. (1973). *Inorg. Chem.* **12**, 272–276.

[bb4] Sheldrick, G. M. (2008). *Acta Cryst.* A**64**, 112–122. 10.1107/S0108767307043930 18156677

[bb5] Westrip, S. P. (2010). *J. Appl. Cryst.* **43**, 920–925.

